# (3*E*,5*E*)-1-Acryloyl-3,5-bis­(2-chloro­benzyl­idene)piperidin-4-one

**DOI:** 10.1107/S1600536811015042

**Published:** 2011-04-29

**Authors:** Alireza Basiri, Vikneswaran Murugaiyah, Hasnah Osman, Madhukar Hemamalini, Hoong-Kun Fun

**Affiliations:** a School of Pharmaceutical Sciences, Universiti Sains Malaysia, 11800 USM, Penang, Malaysia; b School of Chemical Sciences, Universiti Sains Malaysia, 11800 USM, Penang, Malaysia; c X-ray Crystallography Unit, School of Physics, Universiti Sains Malaysia, 11800 USM, Penang, Malaysia

## Abstract

In the title compound, C_22_H_17_Cl_2_NO_2_, the asymmetric unit consists of two crystallographically independent mol­ecules and each piperidinone ring adopts an envelope conformation. The dihedral angles between the two chloro­benzene rings are 24.81 (10) and 19.15 (8)° in the two mol­ecules. In the crystal, mol­ecules are connected *via* weak inter­molecular C—H⋯O hydrogen bonds forming layers perpendicular to the *a* axis.

## Related literature

For details and applications of α,β-unsaturated ketones, see: Anke *et al.* (1981 ▶); Khodair *et al.* (1997 ▶); El-Subbagh *et al.* (2000 ▶); Al-Obaid *et al.* (1996 ▶); El-Barbary *et al.* (1994 ▶); Rungeler *et al.* (1999 ▶); Dimmock *et al.* (1983 ▶). For preparation details of 3,5-bis­(2-chloro­benzyl­idene)piperidin-4-one, see: Dimmock *et al.* (2000 ▶). For ring conformations, see: Cremer & Pople (1975 ▶). For bond-length data, see: Allen *et al.* (1987 ▶). For the stability of the temperature controller used in the data collection, see: Cosier & Glazer (1986 ▶).
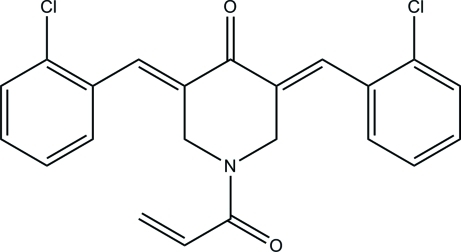


## Experimental

### 

#### Crystal data

C_22_H_17_Cl_2_NO_2_
*M* *_r_* = 398.27
Triclinic, 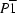

*a* = 9.1426 (5) Å
*b* = 14.3459 (8) Å
*c* = 16.6637 (9) Å
α = 108.348 (1)°
β = 102.695 (1)°
γ = 103.649 (1)°
*V* = 1910.71 (18) Å^3^
*Z* = 4
Mo *K*α radiation
μ = 0.36 mm^−1^
*T* = 100 K
0.44 × 0.40 × 0.16 mm

#### Data collection

Bruker APEXII DUO CCD area-detector diffractometer
Absorption correction: multi-scan (*SADABS*; Bruker, 2009 ▶) *T*_min_ = 0.858, *T*_max_ = 0.947
30297 measured reflections
11008 independent reflections
8952 reflections with *I* > 2σ(*I*)
*R*_int_ = 0.025

#### Refinement

*R*[*F* ^2^ > 2σ(*F* ^2^)] = 0.046
*wR*(*F* ^2^) = 0.132
*S* = 1.03
11008 reflections
503 parameters
H atoms treated by a mixture of independent and constrained refinement
Δρ_max_ = 1.11 e Å^−3^
Δρ_min_ = −0.65 e Å^−3^

### 

Data collection: *APEX2* (Bruker, 2009 ▶); cell refinement: *SAINT* (Bruker, 2009 ▶); data reduction: *SAINT*; program(s) used to solve structure: *SHELXTL* (Sheldrick, 2008 ▶); program(s) used to refine structure: *SHELXTL*; molecular graphics: *SHELXTL*; software used to prepare material for publication: *SHELXTL* and *PLATON* (Spek, 2009 ▶).

## Supplementary Material

Crystal structure: contains datablocks global, I. DOI: 10.1107/S1600536811015042/rz2586sup1.cif

Structure factors: contains datablocks I. DOI: 10.1107/S1600536811015042/rz2586Isup2.hkl

Supplementary material file. DOI: 10.1107/S1600536811015042/rz2586Isup3.cml

Additional supplementary materials:  crystallographic information; 3D view; checkCIF report

## Figures and Tables

**Table 1:** Hydrogen-bond geometry (Å, °)

*D*—H⋯*A*	*D*—H	H⋯*A*	*D*⋯*A*	*D*—H⋯*A*
C2*A*—H2*AA*⋯O2*A*^i^	0.93	2.44	3.146 (2)	133
C2*B*—H2*BA*⋯O2*B*^ii^	0.93	2.49	3.385 (2)	161
C21*A*—H21*A*⋯O2*B*^iii^	0.93	2.47	3.180 (2)	133
C22*A*—H22*A*⋯O1*B*^i^	0.96 (3)	2.59 (3)	3.455 (3)	151 (2)
C22*B*—H22*C*⋯O1*A*^iv^	0.94 (3)	2.40 (3)	3.191 (3)	141 (3)

Symmetry codes: (i) 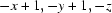; (ii) 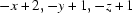; (iii) 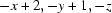; (iv) 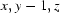.

**Fig. 1.**
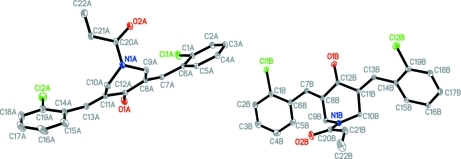
The asymmetric unit of the title compound, showing 30% probability displacement ellipsoids. Hydrogen atoms are omitted for clarity.

**Fig. 2.**
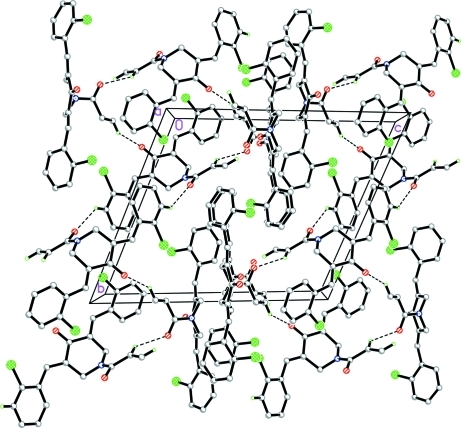
The crystal packing of the title compound viewed along the b axis with hydrogen bonds shown as dashed lines. H atoms not involved in the intermolecular interactions are omitted for clarity.

## supplementary crystallographic information

### Comment

α,β-Unsaturated ketones are a biologically active group of chemicals which are produced by reaction of aldehydes and ketones through Claisen-Schmidt condensation. This class of compounds shows diverse biological activities such as cytotoxic (Anke *et al.*, 1981; Khodair *et al.*, 1997), antitumoral (El-Subbagh *et al.*, 2000; Al-Obaid *et al.*, 1996) and antiviral (El-Barbary *et al.*, 1994) properties. The title compound, (I), includes a conjugated system as the fundamental part in determining the bioactivity of this type of compounds (Rungeler *et al.*, 1999) as well as a β-amino ketone in the structure. These compounds proved to have cytotoxic activities without any mutagenic and carcinogenic side effects (Dimmock *et al.*, 1983).

The asymmetric unit of the title compound consists of two crystallographically independent (3*E*,5*E*)-1-acryloyl- 3,5-bis(2-chlorobenzylidene)piperidin-4-one molecules, (A & B), as shown in Fig. 1.  The bond lengths and angles of molecules A and B agree with each other and are within normal ranges (Allen *et al.*, 1987). The two chlorobenzene rings are inclined to each other forming dihedral angles of 24.81 (10)° (C1A–C6A:C14A–C19A) in molecule A and 19.15 (8)° (C1B–C6B:C14B–C19B) in molecule B.

The piperidine rings (N1A/C8A–C12A and N1B/C8B–C12B) adopt envelope conformations [puckering parameters (Cremer & Pople, 1975): *Q* = 0.5071 (18) Å, θ = 126.1 (2)° and φ = 178.5 (3) °; *Q* = 0.567 (18) Å, θ = 65.89 (18)° and φ = 356.4 (2)°, respectively] with atoms N1A and N1B displaced by 0.6754 (16) and 0.7378 (14) Å from the least-squares plane defined by the remaining atoms (C8A–C12A and C8B–C12B) in the rings.

In the crystal structure (Fig. 2),  the molecules are connected *via* weak intermolecular C2A—H2AA···O2A, C21A—H21A···O2B, C22A—H22A···O1B, C2B—H2BA···O2B and C22B—H22C···O1A hydrogen bonds forming layers perpendicular to the *a* axis.

### Experimental

3,5-Bis(2-chlorobenzylidene)piperidin-4-one was synthesized by the method described by Dimmock *et al.* (2000). Briefly, the title compound (I) was prepared by dropwise addition of an acryloyl chloride solution (8.7 mmol) to a stirred mixture of 3,5-bis(2,4-dichlorobenzylidene)piperidin-4-one (5.8 mmol) and acetone (10 ml) in presence of sodium carbonate (29 mmol) at room temperature. After completion of the reaction (through TLC monitoring), the mixture was poured into ice. The precipitate which formed was filtered and washed with water. The pure solid was then recrystallised from ethanol to afford the title compound as yellow crystals.

### Refinement

Atoms H22A, H22B, H22C and H22D  were located from a difference Fourier map and refined freely. The remaining H atoms were positioned geometrically [C–H = 0.93 or 0.97 Å] and were refined using a riding model, with *U*_iso_(H) = 1.2 *U*_eq_(C).

### Crystal data

**Table:**

C_22_H_17_Cl_2_NO_2_	*Z* = 4
*M**_r_* = 398.27	*F*(000) = 824
Triclinic, *P*1	*D*_x_ = 1.384 Mg m^−^^3^
Hall symbol: -P 1	Mo *K*α radiation, λ = 0.71073 Å
*a* = 9.1426 (5) Å	Cell parameters from 9967 reflections
*b* = 14.3459 (8) Å	θ = 2.6–30.1°
*c* = 16.6637 (9) Å	µ = 0.36 mm^−^^1^
α = 108.348 (1)°	*T* = 100 K
β = 102.695 (1)°	Plate, yellow
γ = 103.649 (1)°	0.44 × 0.40 × 0.16 mm
*V* = 1910.71 (18) Å^3^	

### Data collection

**Table:**

Bruker APEXII DUO CCD area-detector diffractometer	11008 independent reflections
Radiation source: fine-focus sealed tube	8952 reflections with *I* > 2σ(*I*)
graphite	*R*_int_ = 0.025
φ and ω scans	θ_max_ = 30.2°, θ_min_ = 1.6°
Absorption correction: multi-scan (*SADABS*; Bruker, 2009)	*h* = −12→11
*T*_min_ = 0.858, *T*_max_ = 0.947	*k* = −18→20
30297 measured reflections	*l* = −23→23

### Refinement

**Table:**

Refinement on *F*^2^	Primary atom site location: structure-invariant direct methods
Least-squares matrix: full	Secondary atom site location: difference Fourier map
*R*[*F*^2^ > 2σ(*F*^2^)] = 0.046	Hydrogen site location: inferred from neighbouring sites
*wR*(*F*^2^) = 0.132	H atoms treated by a mixture of independent and constrained refinement
*S* = 1.03	*w* = 1/[σ^2^(*F*_o_^2^) + (0.0638*P*)^2^ + 1.181*P*] where *P* = (*F*_o_^2^ + 2*F*_c_^2^)/3
11008 reflections	(Δ/σ)_max_ = 0.001
503 parameters	Δρ_max_ = 1.11 e Å^−^^3^
0 restraints	Δρ_min_ = −0.65 e Å^−^^3^

### Special details

**Table:**

Experimental. The crystal was placed in the cold stream of an Oxford Cryosystems Cobra open-flow nitrogen cryostat (Cosier & Glazer, 1986) operating at 100.0 (1) K.
Geometry. All s.u.'s (except the s.u. in the dihedral angle between two l.s. planes) are estimated using the full covariance matrix. The cell s.u.'s are taken into account individually in the estimation of s.u.'s in distances, angles and torsion angles; correlations between s.u.'s in cell parameters are only used when they are defined by crystal symmetry. An approximate (isotropic) treatment of cell s.u.'s is used for estimating s.u.'s involving l.s. planes.
Refinement. Refinement of F^2^ against ALL reflections. The weighted R-factor wR and goodness of fit S are based on F^2^, conventional R-factors R are based on F, with F set to zero for negative F^2^. The threshold expression of F^2^ > 2σ(F^2^) is used only for calculating R-factors(gt) etc. and is not relevant to the choice of reflections for refinement. R-factors based on F^2^ are statistically about twice as large as those based on F, and R- factors based on ALL data will be even larger.

### Fractional atomic coordinates and isotropic or equivalent isotropic displacement parameters (Å^2^)

**Table:**

	*x*	*y*	*z*	*U*_iso_*/*U*_eq_	
Cl1A	0.73359 (6)	0.69893 (3)	0.19867 (3)	0.03298 (11)	
Cl2A	1.26065 (7)	1.12704 (4)	−0.00037 (5)	0.04944 (15)	
O1A	1.14934 (15)	0.84607 (9)	0.10213 (8)	0.0263 (3)	
O2A	0.62640 (15)	0.62692 (9)	−0.19689 (8)	0.0272 (3)	
N1A	0.89407 (16)	0.67110 (11)	−0.15374 (9)	0.0223 (3)	
C1A	0.71064 (19)	0.57830 (12)	0.11990 (11)	0.0220 (3)	
C2A	0.6011 (2)	0.49154 (14)	0.11782 (12)	0.0272 (3)	
H2AA	0.5427	0.4988	0.1573	0.033*	
C3A	0.5792 (2)	0.39396 (14)	0.05665 (13)	0.0288 (4)	
H3AA	0.5053	0.3355	0.0545	0.035*	
C4A	0.6682 (2)	0.38369 (13)	−0.00161 (12)	0.0269 (3)	
H4AA	0.6551	0.3181	−0.0420	0.032*	
C5A	0.77649 (19)	0.47130 (12)	0.00057 (11)	0.0220 (3)	
H5AA	0.8349	0.4635	−0.0389	0.026*	
C6A	0.79983 (18)	0.57133 (12)	0.06093 (10)	0.0191 (3)	
C7A	0.91519 (18)	0.66502 (12)	0.06621 (10)	0.0182 (3)	
H7AA	0.9626	0.7183	0.1225	0.022*	
C8A	0.96124 (18)	0.68365 (11)	−0.00029 (10)	0.0184 (3)	
C9A	0.8916 (2)	0.61170 (12)	−0.09734 (10)	0.0220 (3)	
H9AA	0.9522	0.5651	−0.1110	0.026*	
H9AB	0.7830	0.5699	−0.1092	0.026*	
C10A	1.05515 (19)	0.73114 (13)	−0.14312 (10)	0.0231 (3)	
H10A	1.0517	0.7655	−0.1848	0.028*	
H10B	1.1166	0.6849	−0.1560	0.028*	
C11A	1.13322 (18)	0.81139 (12)	−0.04866 (10)	0.0189 (3)	
C12A	1.08627 (18)	0.78603 (11)	0.02452 (10)	0.0189 (3)	
C13A	1.23844 (19)	0.90544 (12)	−0.02641 (11)	0.0219 (3)	
H13A	1.2719	0.9500	0.0333	0.026*	
C14A	1.3064 (2)	0.94591 (14)	−0.08475 (12)	0.0293 (4)	
C15A	1.3639 (2)	0.88519 (18)	−0.14667 (13)	0.0379 (5)	
H15A	1.3529	0.8170	−0.1525	0.045*	
C16A	1.4369 (3)	0.9256 (2)	−0.19928 (14)	0.0541 (7)	
H16A	1.4726	0.8843	−0.2405	0.065*	
C17A	1.4558 (3)	1.0263 (2)	−0.18998 (16)	0.0568 (8)	
H17A	1.5055	1.0532	−0.2247	0.068*	
C18A	1.4020 (3)	1.0886 (2)	−0.12963 (18)	0.0535 (7)	
H18A	1.4154	1.1569	−0.1239	0.064*	
C19A	1.3276 (2)	1.04845 (16)	−0.07725 (15)	0.0384 (5)	
C20A	0.7534 (2)	0.67443 (12)	−0.20127 (10)	0.0215 (3)	
C21A	0.7581 (2)	0.73289 (14)	−0.26091 (11)	0.0273 (3)	
H21A	0.8551	0.7700	−0.2623	0.033*	
C22A	0.6255 (3)	0.73198 (17)	−0.31145 (16)	0.0413 (5)	
Cl1B	0.66752 (5)	0.47648 (3)	0.49413 (3)	0.03091 (10)	
Cl2B	0.27364 (6)	−0.25290 (4)	0.23104 (3)	0.03402 (11)	
O1B	0.50001 (14)	0.11375 (9)	0.41521 (8)	0.0239 (2)	
O2B	1.06328 (17)	0.17824 (9)	0.37831 (9)	0.0306 (3)	
N1B	0.92667 (16)	0.09419 (10)	0.44623 (9)	0.0206 (3)	
C1B	0.8476 (2)	0.48776 (12)	0.56386 (11)	0.0226 (3)	
C2B	0.9637 (2)	0.58533 (13)	0.60446 (12)	0.0279 (4)	
H2BA	0.9435	0.6419	0.5943	0.034*	
C3B	1.1093 (2)	0.59741 (14)	0.66010 (12)	0.0326 (4)	
H3BA	1.1891	0.6618	0.6856	0.039*	
C4B	1.1371 (2)	0.51401 (15)	0.67801 (12)	0.0342 (4)	
H4BA	1.2339	0.5231	0.7172	0.041*	
C5B	1.0198 (2)	0.41642 (14)	0.63712 (11)	0.0284 (4)	
H5BA	1.0390	0.3612	0.6503	0.034*	
C6B	0.8727 (2)	0.39952 (12)	0.57631 (10)	0.0213 (3)	
C7B	0.75084 (19)	0.29687 (12)	0.52643 (10)	0.0205 (3)	
H7BA	0.6459	0.2955	0.5128	0.025*	
C8B	0.77430 (18)	0.20415 (12)	0.49812 (10)	0.0186 (3)	
C9B	0.93589 (19)	0.19091 (12)	0.51313 (11)	0.0208 (3)	
H9BA	1.0108	0.2488	0.5098	0.025*	
H9BB	0.9734	0.1904	0.5721	0.025*	
C10B	0.82372 (19)	0.00428 (12)	0.45274 (12)	0.0226 (3)	
H10C	0.8583	0.0066	0.5129	0.027*	
H10D	0.8298	−0.0591	0.4122	0.027*	
C11B	0.65483 (18)	0.00467 (11)	0.42949 (10)	0.0179 (3)	
C12B	0.63143 (18)	0.10822 (12)	0.44444 (10)	0.0182 (3)	
C13B	0.52661 (18)	−0.08136 (12)	0.39480 (10)	0.0184 (3)	
H13B	0.4282	−0.0727	0.3785	0.022*	
C14B	0.52654 (18)	−0.18800 (12)	0.38001 (10)	0.0186 (3)	
C15B	0.63354 (18)	−0.21000 (12)	0.43978 (11)	0.0208 (3)	
H15B	0.7100	−0.1552	0.4892	0.025*	
C16B	0.62897 (19)	−0.31117 (12)	0.42763 (11)	0.0228 (3)	
H16B	0.7017	−0.3234	0.4683	0.027*	
C17B	0.5149 (2)	−0.39402 (12)	0.35426 (11)	0.0244 (3)	
H17B	0.5122	−0.4619	0.3451	0.029*	
C18B	0.4052 (2)	−0.37492 (13)	0.29471 (11)	0.0253 (3)	
H18B	0.3277	−0.4301	0.2460	0.030*	
C19B	0.41142 (19)	−0.27359 (13)	0.30805 (10)	0.0222 (3)	
C20B	0.98641 (19)	0.09578 (12)	0.37874 (11)	0.0211 (3)	
C21B	0.9646 (2)	−0.00569 (14)	0.30854 (12)	0.0308 (4)	
H21B	0.8758	−0.0628	0.2943	0.037*	
C22B	1.0686 (4)	−0.0153 (2)	0.26683 (19)	0.0587 (8)	
H22A	0.620 (3)	0.769 (2)	−0.3504 (17)	0.044 (7)*	
H22C	1.055 (4)	−0.080 (2)	0.223 (2)	0.062 (8)*	
H22B	0.525 (4)	0.697 (2)	−0.3109 (19)	0.058 (8)*	
H22D	1.163 (3)	0.042 (2)	0.2836 (19)	0.055 (8)*	

### Atomic displacement parameters (Å^2^)

**Table:**

	*U*^11^	*U*^22^	*U*^33^	*U*^12^	*U*^13^	*U*^23^
Cl1A	0.0399 (2)	0.0270 (2)	0.0347 (2)	0.00755 (17)	0.02366 (19)	0.00988 (17)
Cl2A	0.0486 (3)	0.0240 (2)	0.0756 (4)	0.0120 (2)	0.0174 (3)	0.0207 (2)
O1A	0.0305 (6)	0.0220 (5)	0.0193 (5)	−0.0006 (5)	0.0088 (5)	0.0050 (4)
O2A	0.0253 (6)	0.0242 (6)	0.0322 (6)	0.0026 (5)	0.0095 (5)	0.0147 (5)
N1A	0.0220 (6)	0.0215 (6)	0.0188 (6)	−0.0016 (5)	0.0039 (5)	0.0102 (5)
C1A	0.0214 (7)	0.0219 (7)	0.0244 (7)	0.0054 (6)	0.0084 (6)	0.0116 (6)
C2A	0.0244 (8)	0.0307 (8)	0.0325 (8)	0.0058 (7)	0.0130 (7)	0.0196 (7)
C3A	0.0251 (8)	0.0245 (8)	0.0379 (9)	0.0020 (6)	0.0080 (7)	0.0193 (7)
C4A	0.0266 (8)	0.0180 (7)	0.0354 (9)	0.0049 (6)	0.0067 (7)	0.0133 (6)
C5A	0.0216 (7)	0.0201 (7)	0.0267 (7)	0.0064 (6)	0.0079 (6)	0.0123 (6)
C6A	0.0181 (7)	0.0201 (7)	0.0220 (7)	0.0056 (5)	0.0060 (5)	0.0126 (6)
C7A	0.0172 (7)	0.0177 (6)	0.0205 (7)	0.0051 (5)	0.0065 (5)	0.0084 (5)
C8A	0.0198 (7)	0.0154 (6)	0.0198 (6)	0.0048 (5)	0.0060 (5)	0.0074 (5)
C9A	0.0261 (8)	0.0172 (7)	0.0195 (7)	0.0027 (6)	0.0045 (6)	0.0081 (5)
C10A	0.0217 (7)	0.0245 (7)	0.0191 (7)	0.0002 (6)	0.0079 (6)	0.0078 (6)
C11A	0.0194 (7)	0.0185 (7)	0.0187 (6)	0.0045 (5)	0.0069 (5)	0.0078 (5)
C12A	0.0203 (7)	0.0168 (6)	0.0210 (7)	0.0050 (5)	0.0086 (5)	0.0083 (5)
C13A	0.0234 (7)	0.0190 (7)	0.0215 (7)	0.0033 (6)	0.0058 (6)	0.0090 (6)
C14A	0.0248 (8)	0.0296 (9)	0.0276 (8)	−0.0008 (7)	0.0026 (6)	0.0146 (7)
C15A	0.0317 (10)	0.0429 (11)	0.0307 (9)	−0.0011 (8)	0.0102 (8)	0.0131 (8)
C16A	0.0311 (11)	0.0887 (19)	0.0284 (10)	−0.0065 (11)	0.0093 (8)	0.0238 (11)
C17A	0.0380 (12)	0.0836 (19)	0.0382 (12)	−0.0107 (12)	0.0044 (9)	0.0379 (13)
C18A	0.0389 (12)	0.0525 (14)	0.0588 (15)	−0.0104 (10)	−0.0047 (10)	0.0409 (12)
C19A	0.0320 (10)	0.0340 (10)	0.0434 (11)	0.0002 (8)	0.0012 (8)	0.0226 (9)
C20A	0.0262 (8)	0.0160 (7)	0.0192 (7)	0.0024 (6)	0.0067 (6)	0.0065 (5)
C21A	0.0305 (9)	0.0257 (8)	0.0259 (8)	0.0033 (7)	0.0080 (7)	0.0152 (7)
C22A	0.0382 (11)	0.0370 (11)	0.0470 (12)	0.0027 (9)	0.0033 (9)	0.0280 (10)
Cl1B	0.0293 (2)	0.0257 (2)	0.0474 (3)	0.01379 (16)	0.01302 (18)	0.02254 (18)
Cl2B	0.0352 (2)	0.0304 (2)	0.0266 (2)	0.00998 (18)	−0.00371 (17)	0.00815 (16)
O1B	0.0216 (6)	0.0246 (6)	0.0301 (6)	0.0109 (4)	0.0074 (5)	0.0148 (5)
O2B	0.0405 (7)	0.0196 (6)	0.0345 (7)	0.0052 (5)	0.0200 (6)	0.0120 (5)
N1B	0.0204 (6)	0.0135 (5)	0.0311 (7)	0.0061 (5)	0.0130 (5)	0.0092 (5)
C1B	0.0270 (8)	0.0208 (7)	0.0267 (7)	0.0106 (6)	0.0143 (6)	0.0117 (6)
C2B	0.0351 (9)	0.0200 (7)	0.0326 (9)	0.0093 (7)	0.0189 (7)	0.0094 (6)
C3B	0.0352 (10)	0.0239 (8)	0.0304 (9)	0.0040 (7)	0.0140 (7)	0.0019 (7)
C4B	0.0348 (10)	0.0313 (9)	0.0263 (8)	0.0113 (8)	0.0046 (7)	0.0012 (7)
C5B	0.0343 (9)	0.0249 (8)	0.0240 (8)	0.0132 (7)	0.0067 (7)	0.0062 (6)
C6B	0.0273 (8)	0.0191 (7)	0.0227 (7)	0.0109 (6)	0.0130 (6)	0.0091 (6)
C7B	0.0231 (7)	0.0206 (7)	0.0251 (7)	0.0104 (6)	0.0118 (6)	0.0130 (6)
C8B	0.0211 (7)	0.0184 (7)	0.0215 (7)	0.0082 (5)	0.0098 (6)	0.0110 (5)
C9B	0.0211 (7)	0.0174 (7)	0.0251 (7)	0.0081 (6)	0.0084 (6)	0.0078 (6)
C10B	0.0189 (7)	0.0167 (7)	0.0375 (9)	0.0062 (5)	0.0116 (6)	0.0153 (6)
C11B	0.0200 (7)	0.0181 (6)	0.0216 (7)	0.0084 (5)	0.0093 (5)	0.0122 (5)
C12B	0.0210 (7)	0.0189 (7)	0.0205 (7)	0.0085 (5)	0.0096 (5)	0.0117 (5)
C13B	0.0190 (7)	0.0200 (7)	0.0196 (6)	0.0076 (5)	0.0070 (5)	0.0104 (5)
C14B	0.0182 (7)	0.0182 (7)	0.0218 (7)	0.0053 (5)	0.0086 (5)	0.0095 (5)
C15B	0.0185 (7)	0.0181 (7)	0.0263 (7)	0.0037 (5)	0.0068 (6)	0.0113 (6)
C16B	0.0210 (7)	0.0202 (7)	0.0313 (8)	0.0068 (6)	0.0095 (6)	0.0145 (6)
C17B	0.0279 (8)	0.0172 (7)	0.0315 (8)	0.0077 (6)	0.0140 (7)	0.0105 (6)
C18B	0.0280 (8)	0.0192 (7)	0.0239 (7)	0.0042 (6)	0.0081 (6)	0.0049 (6)
C19B	0.0225 (7)	0.0237 (7)	0.0201 (7)	0.0074 (6)	0.0063 (6)	0.0087 (6)
C20B	0.0203 (7)	0.0175 (7)	0.0253 (7)	0.0056 (6)	0.0081 (6)	0.0081 (6)
C21B	0.0358 (10)	0.0205 (8)	0.0293 (8)	0.0056 (7)	0.0095 (7)	0.0039 (6)
C22B	0.079 (2)	0.0316 (11)	0.0617 (16)	0.0099 (12)	0.0490 (15)	0.0015 (11)

### Geometric parameters (Å, °)

**Table:**

Cl1A—C1A	1.7434 (17)	Cl1B—C1B	1.7328 (18)
Cl2A—C19A	1.746 (3)	Cl2B—C19B	1.7399 (17)
O1A—C12A	1.2180 (19)	O1B—C12B	1.2244 (19)
O2A—C20A	1.230 (2)	O2B—C20B	1.2283 (19)
N1A—C20A	1.374 (2)	N1B—C20B	1.358 (2)
N1A—C9A	1.454 (2)	N1B—C9B	1.4508 (19)
N1A—C10A	1.460 (2)	N1B—C10B	1.4565 (19)
C1A—C2A	1.387 (2)	C1B—C2B	1.389 (2)
C1A—C6A	1.403 (2)	C1B—C6B	1.407 (2)
C2A—C3A	1.385 (3)	C2B—C3B	1.382 (3)
C2A—H2AA	0.9300	C2B—H2BA	0.9300
C3A—C4A	1.393 (3)	C3B—C4B	1.385 (3)
C3A—H3AA	0.9300	C3B—H3BA	0.9300
C4A—C5A	1.389 (2)	C4B—C5B	1.394 (3)
C4A—H4AA	0.9300	C4B—H4BA	0.9300
C5A—C6A	1.403 (2)	C5B—C6B	1.409 (2)
C5A—H5AA	0.9300	C5B—H5BA	0.9300
C6A—C7A	1.464 (2)	C6B—C7B	1.466 (2)
C7A—C8A	1.347 (2)	C7B—C8B	1.350 (2)
C7A—H7AA	0.9300	C7B—H7BA	0.9300
C8A—C12A	1.501 (2)	C8B—C12B	1.504 (2)
C8A—C9A	1.508 (2)	C8B—C9B	1.510 (2)
C9A—H9AA	0.9700	C9B—H9BA	0.9700
C9A—H9AB	0.9700	C9B—H9BB	0.9700
C10A—C11A	1.513 (2)	C10B—C11B	1.509 (2)
C10A—H10A	0.9700	C10B—H10C	0.9700
C10A—H10B	0.9700	C10B—H10D	0.9700
C11A—C13A	1.341 (2)	C11B—C13B	1.342 (2)
C11A—C12A	1.497 (2)	C11B—C12B	1.502 (2)
C13A—C14A	1.456 (2)	C13B—C14B	1.471 (2)
C13A—H13A	0.9300	C13B—H13B	0.9300
C14A—C19A	1.399 (3)	C14B—C19B	1.401 (2)
C14A—C15A	1.410 (3)	C14B—C15B	1.401 (2)
C15A—C16A	1.395 (3)	C15B—C16B	1.390 (2)
C15A—H15A	0.9300	C15B—H15B	0.9300
C16A—C17A	1.367 (4)	C16B—C17B	1.391 (2)
C16A—H16A	0.9300	C16B—H16B	0.9300
C17A—C18A	1.382 (4)	C17B—C18B	1.389 (2)
C17A—H17A	0.9300	C17B—H17B	0.9300
C18A—C19A	1.396 (3)	C18B—C19B	1.385 (2)
C18A—H18A	0.9300	C18B—H18B	0.9300
C20A—C21A	1.490 (2)	C20B—C21B	1.488 (2)
C21A—C22A	1.310 (3)	C21B—C22B	1.303 (3)
C21A—H21A	0.9300	C21B—H21B	0.9300
C22A—H22A	0.96 (3)	C22B—H22C	0.95 (3)
C22A—H22B	0.94 (3)	C22B—H22D	0.95 (3)
			
C20A—N1A—C9A	119.33 (13)	C20B—N1B—C9B	120.01 (13)
C20A—N1A—C10A	127.79 (14)	C20B—N1B—C10B	127.25 (14)
C9A—N1A—C10A	112.45 (13)	C9B—N1B—C10B	111.67 (13)
C2A—C1A—C6A	122.26 (15)	C2B—C1B—C6B	122.55 (16)
C2A—C1A—Cl1A	117.47 (13)	C2B—C1B—Cl1B	117.54 (13)
C6A—C1A—Cl1A	120.27 (12)	C6B—C1B—Cl1B	119.91 (13)
C3A—C2A—C1A	119.58 (16)	C3B—C2B—C1B	119.30 (17)
C3A—C2A—H2AA	120.2	C3B—C2B—H2BA	120.3
C1A—C2A—H2AA	120.2	C1B—C2B—H2BA	120.3
C2A—C3A—C4A	119.82 (15)	C2B—C3B—C4B	120.32 (17)
C2A—C3A—H3AA	120.1	C2B—C3B—H3BA	119.8
C4A—C3A—H3AA	120.1	C4B—C3B—H3BA	119.8
C5A—C4A—C3A	120.05 (16)	C3B—C4B—C5B	119.92 (18)
C5A—C4A—H4AA	120.0	C3B—C4B—H4BA	120.0
C3A—C4A—H4AA	120.0	C5B—C4B—H4BA	120.0
C4A—C5A—C6A	121.48 (16)	C4B—C5B—C6B	121.53 (17)
C4A—C5A—H5AA	119.3	C4B—C5B—H5BA	119.2
C6A—C5A—H5AA	119.3	C6B—C5B—H5BA	119.2
C5A—C6A—C1A	116.79 (14)	C1B—C6B—C5B	116.21 (15)
C5A—C6A—C7A	122.87 (14)	C1B—C6B—C7B	120.32 (15)
C1A—C6A—C7A	120.29 (14)	C5B—C6B—C7B	123.47 (15)
C8A—C7A—C6A	128.07 (14)	C8B—C7B—C6B	127.12 (15)
C8A—C7A—H7AA	116.0	C8B—C7B—H7BA	116.4
C6A—C7A—H7AA	116.0	C6B—C7B—H7BA	116.4
C7A—C8A—C12A	116.92 (13)	C7B—C8B—C12B	118.21 (14)
C7A—C8A—C9A	124.88 (14)	C7B—C8B—C9B	124.09 (14)
C12A—C8A—C9A	118.15 (13)	C12B—C8B—C9B	117.63 (13)
N1A—C9A—C8A	110.41 (12)	N1B—C9B—C8B	109.99 (13)
N1A—C9A—H9AA	109.6	N1B—C9B—H9BA	109.7
C8A—C9A—H9AA	109.6	C8B—C9B—H9BA	109.7
N1A—C9A—H9AB	109.6	N1B—C9B—H9BB	109.7
C8A—C9A—H9AB	109.6	C8B—C9B—H9BB	109.7
H9AA—C9A—H9AB	108.1	H9BA—C9B—H9BB	108.2
N1A—C10A—C11A	109.79 (12)	N1B—C10B—C11B	109.53 (13)
N1A—C10A—H10A	109.7	N1B—C10B—H10C	109.8
C11A—C10A—H10A	109.7	C11B—C10B—H10C	109.8
N1A—C10A—H10B	109.7	N1B—C10B—H10D	109.8
C11A—C10A—H10B	109.7	C11B—C10B—H10D	109.8
H10A—C10A—H10B	108.2	H10C—C10B—H10D	108.2
C13A—C11A—C12A	117.63 (14)	C13B—C11B—C12B	118.89 (14)
C13A—C11A—C10A	124.21 (14)	C13B—C11B—C10B	124.03 (14)
C12A—C11A—C10A	118.12 (13)	C12B—C11B—C10B	117.06 (13)
O1A—C12A—C11A	120.96 (14)	O1B—C12B—C11B	120.86 (14)
O1A—C12A—C8A	120.93 (14)	O1B—C12B—C8B	121.61 (14)
C11A—C12A—C8A	118.10 (13)	C11B—C12B—C8B	117.52 (13)
C11A—C13A—C14A	127.59 (15)	C11B—C13B—C14B	126.35 (14)
C11A—C13A—H13A	116.2	C11B—C13B—H13B	116.8
C14A—C13A—H13A	116.2	C14B—C13B—H13B	116.8
C19A—C14A—C15A	117.47 (18)	C19B—C14B—C15B	116.55 (14)
C19A—C14A—C13A	121.45 (18)	C19B—C14B—C13B	121.11 (14)
C15A—C14A—C13A	120.93 (17)	C15B—C14B—C13B	122.19 (14)
C16A—C15A—C14A	121.1 (2)	C16B—C15B—C14B	122.09 (15)
C16A—C15A—H15A	119.5	C16B—C15B—H15B	119.0
C14A—C15A—H15A	119.5	C14B—C15B—H15B	119.0
C17A—C16A—C15A	119.8 (3)	C15B—C16B—C17B	119.62 (15)
C17A—C16A—H16A	120.1	C15B—C16B—H16B	120.2
C15A—C16A—H16A	120.1	C17B—C16B—H16B	120.2
C16A—C17A—C18A	120.9 (2)	C18B—C17B—C16B	119.69 (15)
C16A—C17A—H17A	119.6	C18B—C17B—H17B	120.2
C18A—C17A—H17A	119.6	C16B—C17B—H17B	120.2
C17A—C18A—C19A	119.7 (2)	C19B—C18B—C17B	119.85 (15)
C17A—C18A—H18A	120.2	C19B—C18B—H18B	120.1
C19A—C18A—H18A	120.2	C17B—C18B—H18B	120.1
C18A—C19A—C14A	121.1 (2)	C18B—C19B—C14B	122.17 (15)
C18A—C19A—Cl2A	119.90 (19)	C18B—C19B—Cl2B	118.41 (13)
C14A—C19A—Cl2A	119.04 (16)	C14B—C19B—Cl2B	119.40 (13)
O2A—C20A—N1A	120.45 (15)	O2B—C20B—N1B	120.88 (14)
O2A—C20A—C21A	120.99 (16)	O2B—C20B—C21B	121.09 (15)
N1A—C20A—C21A	118.50 (14)	N1B—C20B—C21B	117.92 (14)
C22A—C21A—C20A	119.99 (17)	C22B—C21B—C20B	120.63 (19)
C22A—C21A—H21A	120.0	C22B—C21B—H21B	119.7
C20A—C21A—H21A	120.0	C20B—C21B—H21B	119.7
C21A—C22A—H22A	124.2 (16)	C21B—C22B—H22C	120.4 (18)
C21A—C22A—H22B	122.6 (18)	C21B—C22B—H22D	119.8 (17)
H22A—C22A—H22B	113 (2)	H22C—C22B—H22D	120 (2)
			
C6A—C1A—C2A—C3A	0.9 (3)	C6B—C1B—C2B—C3B	−0.7 (3)
Cl1A—C1A—C2A—C3A	−179.72 (14)	Cl1B—C1B—C2B—C3B	−179.57 (13)
C1A—C2A—C3A—C4A	0.6 (3)	C1B—C2B—C3B—C4B	−2.6 (3)
C2A—C3A—C4A—C5A	−1.2 (3)	C2B—C3B—C4B—C5B	2.4 (3)
C3A—C4A—C5A—C6A	0.3 (3)	C3B—C4B—C5B—C6B	1.0 (3)
C4A—C5A—C6A—C1A	1.1 (2)	C2B—C1B—C6B—C5B	3.8 (2)
C4A—C5A—C6A—C7A	178.74 (15)	Cl1B—C1B—C6B—C5B	−177.27 (12)
C2A—C1A—C6A—C5A	−1.7 (2)	C2B—C1B—C6B—C7B	−175.17 (15)
Cl1A—C1A—C6A—C5A	178.93 (12)	Cl1B—C1B—C6B—C7B	3.7 (2)
C2A—C1A—C6A—C7A	−179.43 (15)	C4B—C5B—C6B—C1B	−4.0 (2)
Cl1A—C1A—C6A—C7A	1.2 (2)	C4B—C5B—C6B—C7B	175.01 (16)
C5A—C6A—C7A—C8A	32.4 (3)	C1B—C6B—C7B—C8B	147.74 (16)
C1A—C6A—C7A—C8A	−150.03 (17)	C5B—C6B—C7B—C8B	−31.2 (3)
C6A—C7A—C8A—C12A	−178.17 (14)	C6B—C7B—C8B—C12B	−177.54 (14)
C6A—C7A—C8A—C9A	4.7 (3)	C6B—C7B—C8B—C9B	−0.6 (3)
C20A—N1A—C9A—C8A	−110.40 (16)	C20B—N1B—C9B—C8B	105.76 (16)
C10A—N1A—C9A—C8A	62.70 (18)	C10B—N1B—C9B—C8B	−63.28 (17)
C7A—C8A—C9A—N1A	147.54 (16)	C7B—C8B—C9B—N1B	−156.07 (14)
C12A—C8A—C9A—N1A	−29.6 (2)	C12B—C8B—C9B—N1B	20.90 (18)
C20A—N1A—C10A—C11A	109.04 (17)	C20B—N1B—C10B—C11B	−101.86 (18)
C9A—N1A—C10A—C11A	−63.35 (18)	C9B—N1B—C10B—C11B	66.19 (17)
N1A—C10A—C11A—C13A	−146.70 (16)	N1B—C10B—C11B—C13B	152.27 (15)
N1A—C10A—C11A—C12A	31.1 (2)	N1B—C10B—C11B—C12B	−26.11 (19)
C13A—C11A—C12A—O1A	−4.7 (2)	C13B—C11B—C12B—O1B	−9.4 (2)
C10A—C11A—C12A—O1A	177.33 (16)	C10B—C11B—C12B—O1B	169.05 (14)
C13A—C11A—C12A—C8A	176.31 (15)	C13B—C11B—C12B—C8B	169.27 (13)
C10A—C11A—C12A—C8A	−1.6 (2)	C10B—C11B—C12B—C8B	−12.25 (19)
C7A—C8A—C12A—O1A	4.4 (2)	C7B—C8B—C12B—O1B	10.9 (2)
C9A—C8A—C12A—O1A	−178.23 (15)	C9B—C8B—C12B—O1B	−166.22 (14)
C7A—C8A—C12A—C11A	−176.63 (14)	C7B—C8B—C12B—C11B	−167.75 (13)
C9A—C8A—C12A—C11A	0.7 (2)	C9B—C8B—C12B—C11B	15.10 (19)
C12A—C11A—C13A—C14A	177.29 (17)	C12B—C11B—C13B—C14B	−176.59 (14)
C10A—C11A—C13A—C14A	−4.9 (3)	C10B—C11B—C13B—C14B	5.0 (2)
C11A—C13A—C14A—C19A	139.06 (19)	C11B—C13B—C14B—C19B	−147.10 (16)
C11A—C13A—C14A—C15A	−45.5 (3)	C11B—C13B—C14B—C15B	37.4 (2)
C19A—C14A—C15A—C16A	−0.9 (3)	C19B—C14B—C15B—C16B	1.5 (2)
C13A—C14A—C15A—C16A	−176.49 (18)	C13B—C14B—C15B—C16B	177.25 (14)
C14A—C15A—C16A—C17A	1.1 (3)	C14B—C15B—C16B—C17B	−0.2 (2)
C15A—C16A—C17A—C18A	−0.6 (4)	C15B—C16B—C17B—C18B	−1.1 (2)
C16A—C17A—C18A—C19A	0.1 (4)	C16B—C17B—C18B—C19B	0.9 (2)
C17A—C18A—C19A—C14A	0.0 (3)	C17B—C18B—C19B—C14B	0.5 (3)
C17A—C18A—C19A—Cl2A	179.58 (17)	C17B—C18B—C19B—Cl2B	179.08 (13)
C15A—C14A—C19A—C18A	0.4 (3)	C15B—C14B—C19B—C18B	−1.7 (2)
C13A—C14A—C19A—C18A	175.93 (18)	C13B—C14B—C19B—C18B	−177.44 (15)
C15A—C14A—C19A—Cl2A	−179.19 (15)	C15B—C14B—C19B—Cl2B	179.76 (12)
C13A—C14A—C19A—Cl2A	−3.6 (3)	C13B—C14B—C19B—Cl2B	4.0 (2)
C9A—N1A—C20A—O2A	−0.7 (2)	C9B—N1B—C20B—O2B	7.7 (2)
C10A—N1A—C20A—O2A	−172.58 (15)	C10B—N1B—C20B—O2B	174.88 (16)
C9A—N1A—C20A—C21A	−178.06 (14)	C9B—N1B—C20B—C21B	−176.05 (15)
C10A—N1A—C20A—C21A	10.0 (2)	C10B—N1B—C20B—C21B	−8.9 (2)
O2A—C20A—C21A—C22A	−1.8 (3)	O2B—C20B—C21B—C22B	26.4 (3)
N1A—C20A—C21A—C22A	175.60 (19)	N1B—C20B—C21B—C22B	−149.8 (2)

### Hydrogen-bond geometry (Å, °)

**Table:**

*D*—H···*A*	*D*—H	H···*A*	*D*···*A*	*D*—H···*A*
C2A—H2AA···O2A^i^	0.93	2.44	3.146 (2)	133.
C2B—H2BA···O2B^ii^	0.93	2.49	3.385 (2)	161.
C21A—H21A···O2B^iii^	0.93	2.47	3.180 (2)	133.
C22A—H22A···O1B^i^	0.96 (3)	2.59 (3)	3.455 (3)	151 (2)
C22B—H22C···O1A^iv^	0.94 (3)	2.40 (3)	3.191 (3)	141 (3)

Symmetry codes: (i) −*x*+1, −*y*+1, −*z*; (ii) −*x*+2, −*y*+1, −*z*+1; (iii) −*x*+2, −*y*+1, −*z*; (iv) *x*, *y*−1, *z*.
